# Genetically driven brain serotonin deficiency facilitates panic-like escape behavior in mice

**DOI:** 10.1038/tp.2017.209

**Published:** 2017-10-03

**Authors:** J Waider, S Popp, M D Lange, R Kern, J F Kolter, J Kobler, N C Donner, K R Lowe, J H Malzbender, C J Brazell, M R Arnold, B Aboagye, A Schmitt-Böhrer, C A Lowry, H C Pape, K P Lesch

**Affiliations:** 1 Division of Molecular Psychiatry, Laboratory of Translational Neuroscience, Department of Psychiatry, Psychosomatics and Psychotherapy, Center of Mental Health, University of Wuerzburg, Wuerzburg, Germany; 2 Institute of Physiology I, Westfaelische Wilhelms-Universität Muenster, Muenster, Germany; 3 Center of Mental Health, Department of Psychiatry, Psychosomatics and Psychotherapy, University of Wuerzburg, Wuerzburg, Germany; 4 Department of Integrative Physiology and Center for Neuroscience, University of Colorado Boulder, Boulder, CO, USA; 5 Laboratory of Psychiatric Neurobiology, Institute of Molecular Medicine, I.M. Sechenov First Moscow State Medical University, Moscow, Russia; 6 Department of Psychiatry and Psychology, School for Mental Health and Neuroscience (MHeNS), Maastricht University, Maastricht, The Netherlands

## Abstract

Multiple lines of evidence implicate brain serotonin (5-hydroxytryptamine; 5-HT) system dysfunction in the pathophysiology of stressor-related and anxiety disorders. Here we investigate the influence of constitutively deficient 5-HT synthesis on stressor-related anxiety-like behaviors using Tryptophan hydroxylase 2 (*Tph2*) mutant mice. Functional assessment of c-Fos after associated foot shock, electrophysiological recordings of GABAergic synaptic transmission, differential expression of the *Slc6a4* gene in serotonergic neurons were combined with locomotor and anxiety-like measurements in different contextual settings. Our findings indicate that constitutive *Tph2* inactivation and consequential lack of 5-HT synthesis in *Tph2* null mutant mice (*Tph2*^*−/−*^) results in increased freezing to associated foot shock and a differential c-Fos activity pattern in the basolateral complex of the amygdala. This is accompanied by altered GABAergic transmission as observed by recordings of inhibitory postsynaptic currents on principal neurons in the basolateral nucleus, which may explain increased fear associated with hyperlocomotion and escape-like responses in aversive inescapable contexts. In contrast, lifelong 5-HT deficiency as observed in *Tph2* heterozygous mice (*Tph*^*+/*^^−^) is able to be compensated through reduced GABAergic transmission in the basolateral nucleus of the amygdala based on *Slc6a4* mRNA upregulation in subdivisions of dorsal raphe neurons. This results in increased activity of the basolateral nucleus of the amygdala due to associated foot shock. In conclusion, our results reflect characteristic syndromal dimensions of panic disorder and agoraphobia. Thus, constitutive lack of 5-HT synthesis influence the risk for anxiety- and stressor-related disorders including panic disorder and comorbid agoraphobia through the absence of GABAergic-dependent compensatory mechanisms in the basolateral nucleus of the amygdala.

## Introduction

Anxiety- and stressor-related disorders represent the most common psychiatric disorders in the European Union with a 12-month prevalence estimate of 14 and 2% of the population.^1^ The neural mechanisms underlying the manifestation of anxiety disorders are complex. However, in patients with anxiety disorders, the extended amygdala^2, 3^ and midbrain serotonergic systems^4, 5^ are commonly dysregulated.

Fear is an acute reaction to a real or perceived immediate threat^6^ and it quickly fades as soon as the threat is removed.^7^ In rodents, acute fear involves the preparation of the animal for fight-or-flight responses. The activation of this behavioral system manifests in complete motionlessness known as freezing.^8, 9^ The brain serotonin (5-hydroxytryptamine; 5-HT) system is thought to play an essential role in the control of anxiety-, fear- and panic-like responses in rodents.^10^ In humans, several lines of evidence link alterations in 5-HT signaling to panic attacks through a defensive behavioral system activated by acute threats.^11^ Panic attacks represent abrupt surges of intense fear or extreme discomfort that reach a peak within minutes. Unexpected panic attacks with at least one of the attacks followed by persistent concern or worry about additional panic attacks or their consequences define panic disorder (PD).^12^ Maladaptive changes in behavior related to the attack, such as agoraphobia, describe anticipatory anxiety and/or marked fear about apparently threatening situations.

5-HT may influence anxiety-, fear- and panic-like responses within the basolateral complex of the amygdala (BLC). In humans, alteration in the Tryptophan hydroxylase 2 gene (*TPH2*) were associated with alterations in amygdala responsiveness to anxiety-related stimuli^13^ as well as anxiety^14^ and panic disorders.^15^ This has been linked recently to alterations in the GABAergic system.^16^ In mice, pharmacological depletion of 5-HT in the BLC reduces anxiety and interferes with fear conditioning.^17^ Furthermore, stress-induced enhancement of fear memory is dependent on 5-HT action within the BLC.^18^ Previously, we provided evidence for increased fear learning in mice deficient for brain 5-HT synthesis, that is (*Tph2*) null mutant mice,^19^ which might be linked to altered 5-HT-receptor function in the BLC.^20, 21^ Together with altered GABA levels, this points toward compensatory changes in the BLC probably mediated through the GABAergic system.^22^ In an attempt to investigate the complex regulatory role of 5-HT on fear and anxiety, this study evaluates fear-related behaviors in null mutant (*Tph2*^−^^/−^), heterozygous (*Tph2*^+/−^) and wild-type (*Tph2*^+/+^) mice in association with functional neuroanatomical approaches in the BLC and expression of 5-HT-related genes in serotonergic neurons within the brainstem raphe nuclei. These experiments were complemented by recordings of inhibitory synaptic activity in the BLC in order to assess the functional impact on the GABAergic system.

## Materials and methods

### Behavior

Male *Tph2*^+/+^, *Tph2*^+/−^ and *Tph2*^−^^/−^ mice, aged 2–6 months, were housed individually in a controlled environment (12/12 h light/dark cycle, 21±0.5 °C room temperature, 50±5% humidity) with food and water *ad libitum* unless stated otherwise.

Mice were acclimatized to the housing conditions for ⩾1 week prior to behavioral experiments. Tests were performed during the light phase between 1000 and 1500 hours with a recovery period of ⩾3 days between different tests. Naive mice (*n*=11/genotype) were first tested for anxiety-like behavior in the light–dark transition test (LDT) followed by an open-field test (OFT) to assess locomotor activity in a novel environment and a two-trial social interaction test (SIT) to determine sociability and preference for social novelty. After a recovery period of 10 days, a subset of these mice (*n*=7/genotype) underwent home cage activity testing (HCT) to measure locomotor activity in a familiar environment. A second cohort of naive mice (*n*=10–11/genotype) was tested for anxiety-/depression-like behavior in the novelty-suppressed feeding test (NSFT) and compulsive-like digging behavior in the marble burying test (MBT).

**Figure 1:**
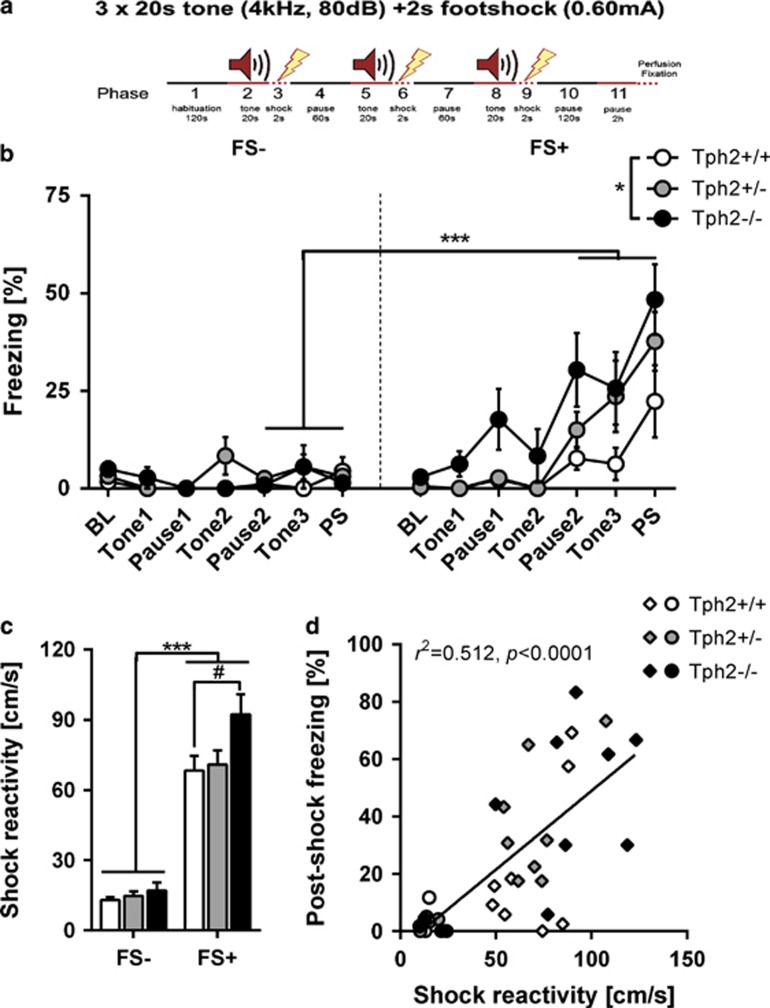
Increased freezing and shock reactivity in Tph2-deficient mice. Paired foot shock (FS) protocol (**a**). Mice were exposed to a novel context for 120 s. After 120 s, a tone was paired with a FS and repeated two times with a 60 s pause. Mice were returned to the home cage 120 s after the last FS and killed 2 h later for c-Fos analysis. Freezing during fear conditioning was compared between FS+ (*n*=8/genotype) and FS− (*n*=3–4/genotype) mice (**b**). Shock reactivity in the FS+ and FS− groups was analyzed (**c**) and correlated with post-shock freezing (**d**). Data are shown as means±s.e.m. (**b**) or +s.e.m. (**c**). ^#^0.1>*P*>0.05, **P<0.01 and ***P<0.001 compared to respective controls.

For fear conditioning including foot shocks (FSs) and subsequent c-Fos immunostaining experiments, animals were randomly assigned to one of the three groups: mice that were left undisturbed in the home cage (Ctrl: *n*=7–11/genotype), mice that were exposed to the FS chamber without receiving foot shocks (FS−: *n*=3–4/genotype) and mice that received three tone+FS pairings followed by immediate (2 h) brain dissection (FS+: *n*=8/genotype). FS were applied in an automated conditioning chamber (TSE Systems, Bad Homburg, Germany). In detail, after a 120 s habituation period to the novel context, mice received three pairings of a 20 s tone (80 dB, 4 kHz) co-terminating with a 2 s FS (0.6 mA) with a 60 s pause between successive tone–shock pairings (Figure 1a). Mice were returned to their home cage 120 s after the last FS.

Freezing behavior (defined as complete immobility for a duration of >2 s) was automatically recorded via infrared light beams. The maximum velocity during FS delivery was used as an index of shock reactivity.

### C-Fos immunostaining

Two hours after the fear conditioning protocol brains were fixed by transcardial perfusion. Serially cut 30 μm cryostat sections were used for immunofluorescent stainings. Primary antibodies used were mouse anti-parvalbumin (PV) (1:200; Swant, Marly, Switzerland) and rabbit anti-c-Fos (1:400; Santa Cruz Biotechnology, Dallas, TX, USA). Sections were incubated in 1:400 diluted secondary antibodies, goat anti-rabbit 555 and goat anti-mouse 488 (Invitrogen, Carlsbad, CA, USA).

For the BLC, lateral (LA) and basolateral nucleus (BL) nucleus of 3–4 consecutive sections from −0.90 mm bregma to −1.82 mm bregma, spaced 180 μm apart, were identified using DAPI (4′, 6-diamidino-2-phenylindole) counterstaining and delineated with contours according to the mouse brain atlas.^23^ Cells were counted as c-Fos-ir only when the nucleus showed complete fluorescent signal. The sum of counted cells of all sections per mouse were divided by the total contour area to calculate the cell densities per region of interest.

### Slc6a4 *in situ* hybridization

Behaviorally naive animals male *Tph2*^+/+^, *Tph2*^+/−^ and *Tph2*^−^^/−^ mice (*n*=6–8/genotype), aged 2–6 months, were housed in groups in a controlled environment (12/12 h light/dark cycle) with food and water *ad libitum* and were killed via rapid decapitation after isoflurane anesthesia. Following decapitation, brains of all experimental mice were removed, snap-frozen on dry ice and stored at −80 °C. Brains were placed on dry ice and shipped to the University of Colorado, Boulder. Brains were dissected into forebrain and hindbrain blocks with a cut in the coronal plane at the caudal border of the mammillary bodies (approximately −3.40 mm bregma) using a mouse brain matrix (RBM-2000C, ASI Instruments, Warren, MI, USA), to ensure a consistent coronal plane of sectioning as described previously.^24^ ^35^S-radiolabeled oligonucleotide probes complementary and specific to *Slc6a4* mRNA (5′-ACTGCAGAGTACCCATTGGATATTTGGCTAGGCTCTGCCCTGTCCGCTGT-3′) (Integrated DNA Technologies, Coralville, IA, USA) were used. After hybridization, slides were air-dried, and apposed onto a BioMax MR autoradiography film (Cat. No. 871 5187, Carestream Health, Rochester, NY, USA) along with ^14^C standards (Cat. No. ARC0146C, American Radiolabeled Chemicals, St Louis, MO, USA) for a period of 36 days (*Slc6a4*).

For semi-quantitative analysis of *Slc6a4* mRNA expression, gray values of digital autoradiography images were analyzed with ImageJ (NIH, Bethesda, MD, USA).

### Electrophysiology

Five- to six-month old behaviorally naive *Tph2*^−/−^, *Tph2*^+/−^ or *Tph2*^+/+^ mice were housed in groups in a controlled environment (12/12 h light/dark cycle) with food and water *ad libitum*. Mice were anesthetized with Forene (isoflurane, 1-chloro-2,2,2-trifluoroethyl-difluoromethyl ether; 2.5%) and decapitated. Coronal slices (300 μm thickness) containing the amygdala were prepared on a vibratome (Leica VT1200S, Wetzlar, Germany), incubated at 32 °C for 20 min and stored thereafter at room temperature (RT). Single slices were placed at RT in a submersion chamber and were perfused with artificial-cerebrospinal fluid (ACSF) containing (in mM): NaCl 120, KCl 2.5, NaH_2_PO_4_ 1.25, MgSO_4_ 2, CaCl_2_ 2, NaHCO_3_ 22 and glucose 20. The pH was set to 7.35 by gassing with carbogen. Whole-cell patch-clamp recordings were performed using an EPC-10 patch-clamp amplifier (HEKA, Lambrecht, Germany) at a sampling rate of 10 kHz. The recordings were done on principal neurons in the BL, which were morphologically and electrophysiologically identified as previously described.^25^ Patch pipettes (2.2-2,8 MΩ borosilicate glass; Clark Electromedical Instruments, UK) were filled as follows: (in mM): NaCl 10, KCl 110, EDTA 11, HEPES 10, MgCl_2_ 1, CaCl_2_ 0.5, phosphocreatine 15, MgATP 3 and NaGTP 0.5. The pH was set to 7.25. Inhibitory postsynaptic currents (IPSCs) were recorded in voltage-clamp mode at a membrane potential of −70 mV in the presence of AP-5 (D-(-)-2-amino-5-phosphonopentanoic acid; 50 μM; Abcam, Cambridge, UK) and DNQX (6,7-dinitroquinoxaline-2,3-dione disodium salt; 10 μM; Abcam) to block NMDA- and AMPA receptors, respectively. Spontaneous (s) IPSCs were analyzed over a time period of 5 min, miniature (m) IPSCs were recorded in the presence of tetrodotoxin (TTX, 0.5 μM) and evoked (e) IPSCs were captured after electrical microstimulation (bipolar stimulation electrode placed in the LA local neuropil; 500 μs).

### Statistical analyses

Behavioral data were analyzed using IBM SPSS Statistics 21. For analysis of immunohistochemical staining, total cell densities were evaluated using GraphPad Prism version 6.00 (GraphPad Software, San Diego, CA, USA). Two-way analysis of variance with Tukey’s *post hoc* testing was used if not stated otherwise. For statistical comparisons of the *Slc6a4* expression, the software package SPSS (version 22.0, SPSS, Chicago, IL, USA) was used in a linear-mixed model analysis. For analysis of electrophysiology data, mean frequencies and amplitudes of individual recordings were averaged and significance was determined using unpaired Student’s *t*-test. The number of experiments are given as: no. of cells/no. of animals. Results are presented as mean±s.e.m., unless stated otherwise. The significance level was set at *P*<0.05. The data sets were tested for statistically significant outliers using the Grubbs’ test (significance level *P*<0.05).

For detailed procedures see Supplementary Material

All experiments were performed in accordance with the European Parliament and Council Directive (2010/63/EU) and were approved by local authorities (Wuerzburg: 55.2-2531.01- 57/12; Muenster: LANUV-NRW 8.87-51.05.20.10.218).

## Results

### Fear and escape responses in *Tph2*-deficient mice

Paired FS as used in fear-conditioning protocols has been shown to activate 5-HT neurons and results in a fast release of 5-HT within the BLC.^18^ Furthermore, we previously provided evidence for increased freezing behavior after two tone-signaled FSs in *Tph2*^−/−^ mice.^19^ To investigate the underlying mechanisms in more detail, we applied a modified fear-conditioning paradigm using three tone–shock pairings (FS+ group, Figure 1a). As control for non-associative freezing, an independent group of mice was subjected to the same protocol, but without FSs (FS− group).

Analysis of freezing revealed a significant phase × group interaction (F_(3,99)_=8.59, *P*<0.001; Figure 1b), showing that freezing was significantly increased in FS+ mice from the second FS onwards. Split-group analyses detected significantly elevated freezing (F_(2,21)_=4.28, *P*=0.028; Figure 1b) in FS+ *Tph2*^−/−^ mice compared to FS+ *Tph2*^+/+^ littermates. Furthermore, the motor response evoked by FS was higher in FS+ mice compared to the FS− group (F_(1,29)_=98.42, *P*<0.001; Figure 1c) and positively correlated with post-shock freezing (*r*^2^= 0.521, *P*<0.001; Figure 1d). Split-group analyses detected significantly elevated FS-evoked motor response (F_(2,21)_=3.48, *P*=0.05; Figure 1c) in FS+ *Tph2*^−/−^ mice compared to FS+ *Tph2*^+/+^ littermates of the same group.

### Pattern of c-Fos reactivity in the amygdala

**Figure 2:**
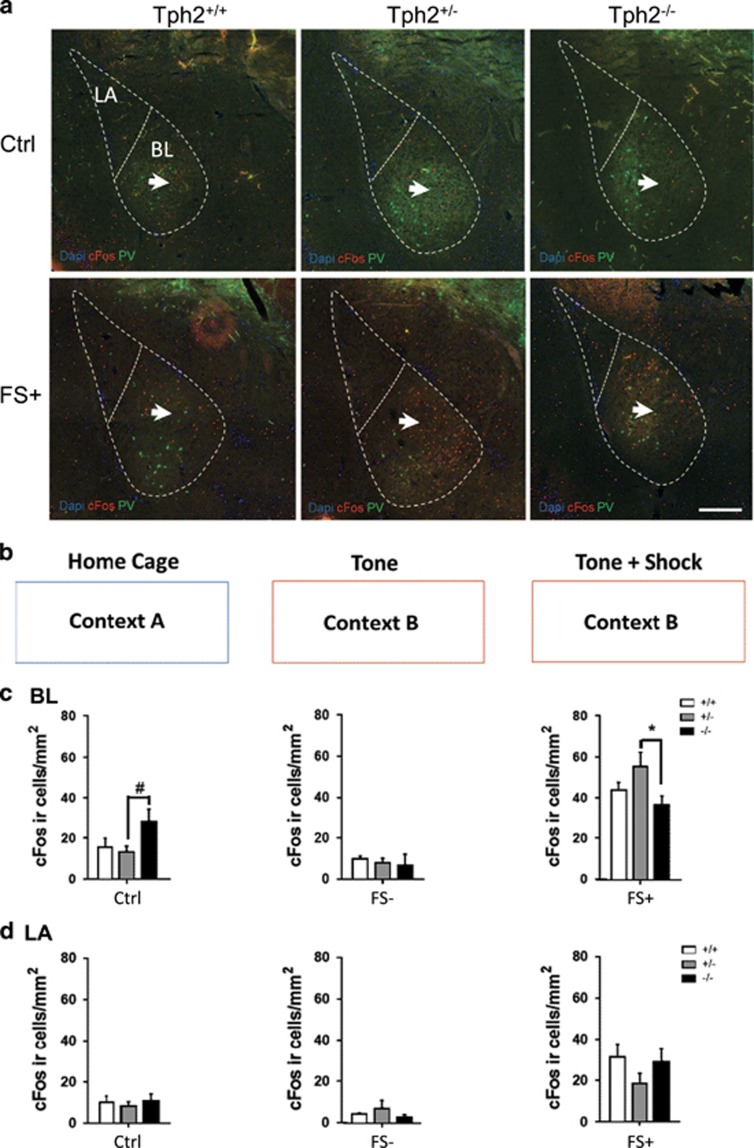
Foot shock differentially activates the basolateral complex of amygdala in *Tph2*-deficient mice. Anti-parvalbumin (PV) (green) and c-Fos (red) immunofluorescent staining with DAPI (blue) of *Tph2*^*+/+*^, *Tph2*^*+/−*^ and *Tph2*^*−/−*^ mice in the lateral (LA) and basolateral (BL) nucleus of the basolateral amygdala (**a**). c-Fos immunostaining was analyzed in *Tph2*^*+/+*^, *Tph2*^*+/*^^−^ and *Tph2*^−^^/−^ mice under home cage control conditions (Ctrl) (*n*=5–7), mice that were placed in the conditioning context but did not receive foot shocks (FS−, *n*=3-4) and after the foot shock presentation (FS+, *n*=5–7/condition) (**b**). c-Fos-immunoreactive cell densities were analyzed between Ctrl, FS− and FS+ cohorts in the BL (**c**) and LA (**d**). Arrows in **a** indicate c-Fos ir cells in the anterior BL. Data are shown as means+s.e.m. ^#^0.1>*P*>0.05, *P<0.05 and compared to respective controls. Scale bar, 100 μm (**a**).

It is well established that the BLC is a key region for fear processing. In order to identify Tph2-dependent pattern of activity within the BLC, mice were killed 2 h after the fear-conditioning protocol for double immunofluorescent staining of c-Fos with parvalbumin (PV; a marker of a subset of GABAergic interneurons). FS increased c-Fos expression in PV-immunonegative cells in the anterior part of the BL (Figure 2a). Further quantification of cell densities in FS+, FS− and a home cage control group (Ctrl) in the LA and BL (Figures 2b and c) detected a genotype × group interaction for the density of c-Fos immunoreactive (ir) neurons in the BL (F_(4,35)_=3.28, *P*=0.022; Figure 2c) and a main group effect (F_(2,35)_=38.51, *P*<0.001) showing that exposure to novel contexts reduced c-Fos (Ctrl vs FS− *P*=0.0317) and FS increased c-Fos-ir in all genotypes (FS− vs FS+ *P*<0.001). Further analysis revealed a tendency toward increased density of c-Fos-ir neurons in Ctrl *Tph2*^−/−^ mice compared to Ctrl *Tph2*^+/−^ (*P*=0.078) (Figure 2d) and a significant increase of c-Fos-ir density in FS+ *Tph2*^+/−^ compared to FS+ *Tph2*^−/−^ mice (*P*=0.028). The results indicate an elevated activation of the BL in*Tph2*^−^^/−^ mice under home cage conditions, which is blocked by exposure to novel contexts. Furthermore, activation of the BL by FS was most pronounced in *Tph2*^+/−^ mice (*P*=0.023 compared Ctrl *Tph2*^+/−^ and <0.001 compared to FS− *Tph2*^+/−^), while in *Tph2*^−^^/−^ c-Fos activation after FS did not exceed c-Fos levels of Ctrl *Tph2*^−^^/−^ mice (*P*=0.92.). Still a significant increase in c-Fos due to FS was detected compared to FS- *Tph2*^−^^/−^ (*P*=0.022). In the LA, analysis of variance detected only a main group effect on the density of c-Fos-ir neurons (F_(2,35)_=19.18, *P*<0.001; Figure 2d). Here the FS+ group was increased compared to Ctrl (*P*<0.001) and FS− (*P*<0.001) showing that FS independent of 5-HT activates the LA.

### Alteration in GABAergic synaptic transmission in the BL

**Figure 3:**
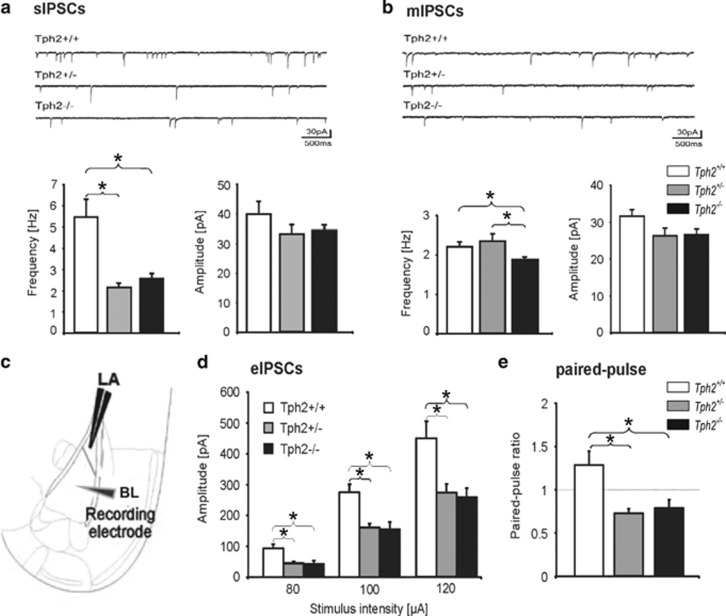
Inhibitory currents in the basolateral nucleus of the amygdala of *Tph2*-deficient mice. Electrophysiological investigation of GABAergic synapse function *in vitro* of naive *Tph2*^−/−^, *Tph2*^+/−^ and *Tph2*^+/+^ mice. (**a**) Upper panel: mean frequency (left) and amplitude (right) of spontaneous inhibitory postsynaptic currents (sIPSCs) in *Tph2*^−/−^ (*n*=21/3)*, Tph2*^*+/*^^−^ (*n*=24/4) and *Tph2*^*+/+*^ (*n*=19/3) mice with example traces shown in the lower panel. (**b**) Upper panel: mean frequency and amplitude of miniature inhibitory postsynaptic currents (mIPSCs) in *Tph2*^−/−^ (*n*=18/4), *Tph2*^*+/−*^ (*n*=18/3) and *Tph2*^*+/+*^ (*n*=16/3) mice with example traces shown in the lower panel. (**c**) Scheme of recording indicating the stimulating electrode in the lateral nucleus (LA) and the patch-clamp pipette in the basolateral nucleus of the amygdala (BL). (**d**) Mean amplitude of evoked inhibitory postsynaptic currents (eIPSCs) recorded at different stimulus intensities in pyramidal neurons from *Tph2*^−/−^, *Tph2*^*+/*^^−^ and *Tph2*^*+/+*^ *mice.* Note significant reduction of eIPSCs at all tested stimulation intensities in *Tph2*^−/−^ (*n*=14/4) and *Tph2*^*+/−*^ (*n*=13/3) compared to wild types (*n*=12/3) as well as paired-pulse ratio (**e**). Data are shown as means+s.e.m. *P<0.05, **P<0.01 and ***P<0.001.

Next, we hypothesized that the observed differences in behavior and the increased activity of the BL in *Tph2^−/−^* mice under home cage control conditions are based on impairments of GABAergic synaptic transmission.^25, 26^ Therefore, IPSCs were recorded under whole-cell voltage-clamp conditions from principal neurons (PNs) in the BL of *Tph2*^−/−^, *Tph2^+/−^*and *Tph2*^*+/+*^ mice. The frequency of spontaneous inhibitory postsynaptic currents (sIPSCs) was reduced in BL PNs of *Tph2*^*+/*^^−^ (*n*=24/4; *P*<0.001) and *Tph2*^*−/−*^ mice (*n*=21/3; *P*<0.001), compared to wild-type littermates (*n*=19/3; Figure 3a), while the amplitude was not affected (Figure 3a). Furthermore, *Tph2*^−/−^ showed a reduced frequency of miniature IPSCs (mIPSCs) (*n*=18/4; *P*<0.01) relative to *Tph2*^*+/+*^ and *Tph2*^*+/−*^ mice (Figure 3b), whereas the mIPSCs frequency of *Tph2*^*+/−*^ (*n*=18/3) was unaltered compared to wild types (*n*=16/3; Figure 3b). Next, GABAergic synaptic transmission was evoked by electrical microstimulation within the LA (Figure 3c). Here, amplitudes of evoked IPSCs (eIPSCs) showed a significant reduction in *Tph2*^*+/−*^ (*n*=13/3; *P*<0.01) and *Tph2*^−/−^ (*n*=14/4; *P*<0.01) compared to *Tph2*^*+/+*^ mice (*n*=12/3) at all tested stimulation intensities (Figure 3d). Paired-pulse facilitation (PPF) was examined next. PPF refers to an increase in a second synaptic response in a double stimulation protocol, relating to a presynaptically mediated increase in transmitter release. Here PPF was determined by applying pairs of electrical pulses separated by 50 ms to the local LA neuropil, and the ratio of IPSCs upon the second and the first stimulation was calculated (Figure 3e). Paired pulse facilitation observed in *Tph2*^*+/+*^ (*n*=12/3) was significantly affected in *Tph2*^*+/−*^ (*n*=13/3; *P*<0.01) and *Tph2*^−/−^ (*n*=14/4; *P*<0.01) (Figure 3e). These findings suggest an impairment of presynaptic release mechanisms in BL PNs of *Tph2*^+/^^−^ and *Tph2*^−/−^ mice.

### Differential expression of *Slc6a4* in the raphe nuclei

**Figure 4:**
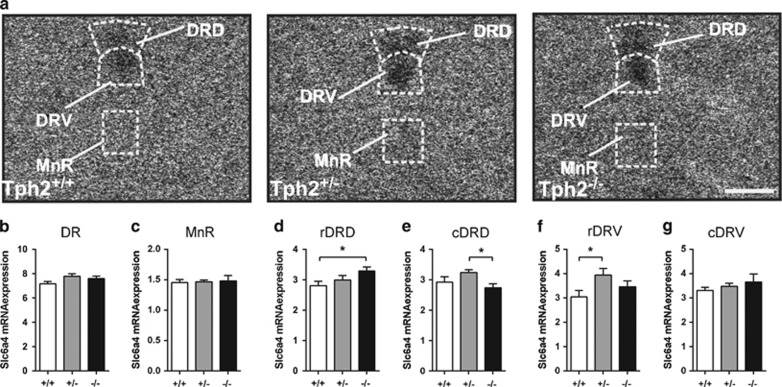
Expression of *Slc6a4* mRNA across the rostrocaudal extent of the dorsal raphe nucleus. (**a**) Photomicrographs illustrating *Slc6a4* (solute carrier family 6, member 4; *5-Htt*) mRNA expression in *Tph2*^*+/+*^ mice (left), *Tph2*^*+/−*^ mice (middle) and *Tph2*^−/−^ mice (right) at −4.364 mm bregma. (**b**–**e**) Mean expression of *Slc6a4* mRNA across the rostrocaudal extent of (**b**) the entire dorsal raphe nucleus (DR) and (**c**) MnR, (**d**) rDRD and (**e**) cDRD as well as (**f**) rDRV and (**g**) cDRV of *Tph2*^*+/+*^, *Tph2*^*+/−*^ and *Tph2*^−/−^ mice (*n*=6–8 per genotype). DR, dorsal raphe nucleus; DRD, dorsal raphe nucleus, dorsal part; DRV, dorsal raphe nucleus, ventral part; MnR, median raphe nucleus. *n*=6–8/genotype; *P<0.01. Scale bar, 200 μm (**a**).

The BLC is strongly innervated by serotonergic fibers of the dorsal raphe nuclei.^27^ Indeed, analysis of 5-HT transporter (*Slc6a4)* mRNA expression (Figure 4a) throughout the dorsal raphe nucleus revealed an overall genotype effect (F_(2,51.7)_=4.3, *P*=0.019; Figure 4b; Supplementary Table S1), which was absent in the median raphe nucleus (Figure 4c). Furthermore, a significant genotype × rostrocaudal level interaction (F_(122,56.2)_=7.1, *P*<0.001; Figure 4b; Supplementary Table S1) was detected. Subregional analyses revealed localized increases in *Slc6a4* mRNA expression in the rostral region of the dorsal raphe nucleus, dorsal part (rDRD) in *Tph2*^−/−^ mice relative to *Tph2*^+/+^ mice (*P*=0.038; Figure 4d; Supplementary Figure S4) and in the caudal region of the dorsal raphe nucleus, dorsal part (cDRD) in *Tph2*^*+/−*^ relative to *Tph2*^−/−^ mice (*P*=0.023; Figure 4e; Supplementary Figure S4). Analysis also revealed localized increases in *Slc6a4* mRNA expression in the rostral, but not caudal, region of the dorsal raphe nucleus, ventral part (rDRV) in *Tph2*^*+/−*^ mice relative to *Tph2*^+/+^ mice (*P*=0.026; Figures 4f and g; Supplementary Figure S4).

### Increased escape-like behaviors accompany hyperlocomotion in Tph2^−/−^ mice

**Figure 5:**
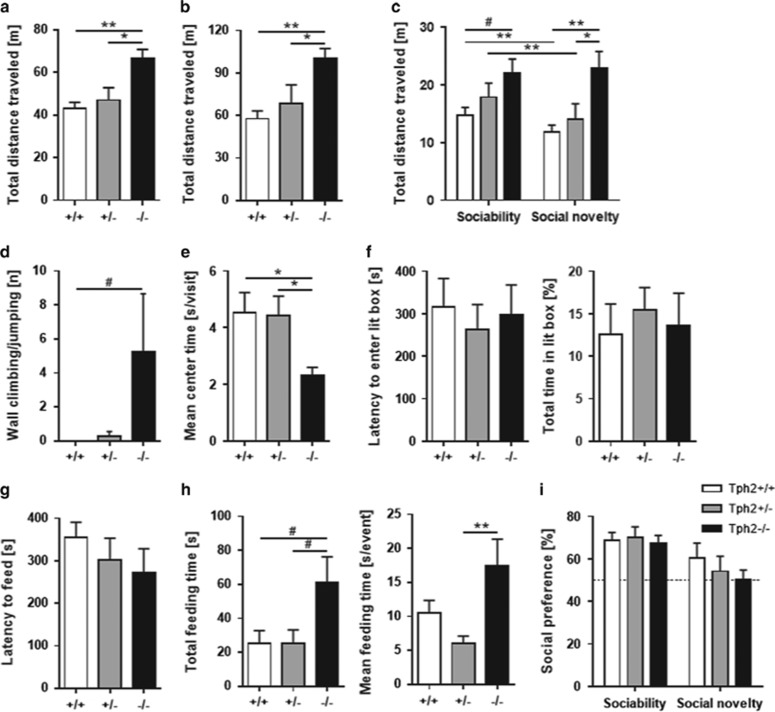
5-HT deficiency induces hyperlocomotion and escape behaviors in different contexts. Locomotor activity of *Tph2*^+/+^, *Tph2*^+/−^ and *Tph2*^−/−^ mice was measured over 30 min in the home cage (*n*=10–11/genotype) and in the open field (*n*=11/genotype) (**a**) as well as in a two-trial social interaction test (**b**) (*n*=7/genotype). Escape responses were detected as wall jumps in the open field (**c**). Further anxiety-like measures (*n*=11/genotype) included mean open-field center time (**d**) as well as latency to enter (**e**) and time in the light compartment of the light–dark box (**f**). Novelty-suppressed feeding (*n*=10–11/genotype) included latency to feed (**g**) as well as total feeding time (**h**). Social behaviors were evaluated using measures of sociability and preference for social novelty (**i**). Data are shown as means+s.e.m. ^#^*P*<0.1, *P<0.05 and **P<0.01 compared to respective controls.

Here, we showed increased fear and an impaired activity pattern of BA neurons in 5-HT-deficient mice to inescapable FS due to an altered BA response involving GABAergic dysregulation. In former studies using *Tph2*^−/−^ mice conflicting results were obtained regarding anxiety-like behaviors,^28, 29, 30^ probably reflecting a confounding locomotion phenotype depending on the aversive nature of the context.^19^ Therefore, a series of behavioral paradigms were conducted to test different aspects of lifelong 5-HT deficiency on locomotor activity and anxiety-like behavior in different contextual settings. Indeed, *Tph2*^−/−^ mice displayed motor abnormalities in several behavioral tests, which manifested as enhanced locomotion and escape behavior in both familiar (HCT, SIT) and novel (OFT, MBT) environments. Specifically, *Tph2*^−/−^ mice traveled significantly longer distances than *Tph2*^+/−^ and *Tph2*^+/+^ littermates in the home cage (HCT: F_(2,18)_=8.63, *P*=0.002, Figure 5a), in the open-field (OFT: F_(2,30)_=5.92, *P*=0.007, Figure 5a, Supplementary Figure S1) and in the social interaction test (SIT: F_(2,29)_=4.53, *P*=0.019, Figure 5b). Additionally, *Tph2*^−/−^ mice exhibited impaired intersession habituation in the SIT (trial × genotype interaction for distance traveled: F_(2,29)_=4.48, *P*=0.020, Figure 5b) and reduced intrasession habituation in the marble burying test (MBT; time × genotype interaction for distance traveled: F_(10.90, 157.56)_=1.87, *P*=0.048, Supplementary Figure S2).

Furthermore, a significant increase in escape-oriented wall-climbing and jumping behavior, which accompanied the hyperactivity phenotype of *Tph2*^−/−^ mice in novel environments, was detected (OFT: *χ*^2^_(2)_ =6.14, *P*=0.046, Figure 5c; MBT: *χ*^2^_(2)_ =13.54, *P*=0.001, Supplementary Figure S2). Moreover, *Tph2*^−/−^ mice spent significantly less time in the center of the open-field compared to *Tph2*^+/−^ and *Tph2*^+/+^ littermates (mean center time per visit: F_(2,30)_=4.47, *P*=0.020, Figure 5d; total center time: F_(2,30)_=2.97, *P*=0.067; Supplementary Figure S1), which together with increased escape behavior, points toward exaggerated anxiety-like behavior in the OFT.

However, other anxiety-related tests did not reveal genotype differences, that is, the number of marbles buried in the MBT (Supplementary Figure S2) as well as the latency to enter and time spent in the lit box in the light–dark transition test (LDT; Figures 5e and f). Interestingly, despite similar latencies to feed in the NSFT (Figure 5g), *Tph2*^−/−^ mice spent significantly more time feeding in the center of the novel, brightly lit arena than their *Tph2*^+/−^ and *Tph2*^+/+^ littermates (total feeding time: F_(2,28)_=3.68, *P*=0.038, Figure 5h; mean feeding time per event: F_(2,28)_=5.33, *P*=0.011, Figure 5h), thereby arguing for reduced anxiety-like behavior in the presence of rewarding stimuli such as food pellets. In the SIT, social preference (stranger mouse vs empty cage in the sociability test and familiar vs novel mouse in the social novelty test; Figure 5i) and the total time spent in the interaction zones (Supplementary Figure S2) did not differ among genotypes. However, as a consequence of their hyperactive phenotype, *Tph2*^−/−^ mice crossed the interaction zones more frequently than *Tph2*^+/−^ and *Tph2*^+/+^ littermates, but spent significantly less time engaged in active contact (mean interaction time per visit: F_(8,54)_=2.40, *P*=0.027; Supplementary Figure S2).

## Discussion

The amygdala is a key brain region for fear processing.^31^ The 5-HT system has been linked to altered responsiveness of the amygdala^13^ and is associated with stressor- and anxiety related disorders^14, 15^ involving GABAergic dysregulation^16^. We believe our findings indicate for the first time that constitutive Tph2 inactivation and consequential lack of 5-HT synthesis in mice results in increased freezing to associated FS and a differential c-Fos activity pattern in the BLC. This is accompanied by altered GABA transmission indicated by IPSC recordings on PNs of the BL. In contrast, a slight 5-HT deficiency in *Tph2*^*+/−*^ mice is able to be compensated through reduced GABAergic transmission on PNs of the BL resulting in increased activity of the BL due to FS, involving *Slc6a4* mRNA upregulation in subdivision of DR neurons.

Under home cage conditions, *Tph2*^−/−^ mice showed increased c-Fos activity patterns of BL neurons compared to *Tph2*^−/−^ and *Tph2*^+/-^ mice, which may be based on the absence of compensation effects, due to complete absence of 5-HT synthesis. However, altered expression of *Slc6a4* in the cDRD and rDRV of *Tph2*^*+/−*^ mice supports previous findings, showing that *Tph2*-deficient mice are characterized by an altered 5-HT metabolism in different brain regions. This involves compensation through 5-HT1a and 5-HT1b receptors^32, 33, 34^ as well as 5-HT2 receptors^20^ in the amygdala. Here we show that mRNA expression is altered in a rostrocaudal fashion,^35^ which implies subgroups of 5-HT neurons involved in *Slc6a4* upregulation in *Tph2*^+/−^ mice.

Exposing mice to novel environments seems to inhibit the increased c-Fos activity detected under home cage control conditions in the BL of *Tph2*^−/−^ mice. During fear conditioning, the hippocampal formation has been shown to be involved in a network of brain structures encoding the contextual component of fear especially during early acquisition phases.^36, 37^ Indeed, novel contexts seem to activate the dorsal hippocampus,^38^ and GABAergic interneurons in the BLC were shown to be activated during hippocampal theta network activity or optogenetic stimulation of CA1 pyramidal neurons, whereas principal neurons in the BLC are inhibited.^39, 40^ This, in turn, may explain the low c-Fos activity in the BL of the FS− group exposed to a novel context.

In contrast, negatively reinforced stimuli have been shown to activate GABAergic neurons in the BLC,^41^ which is probably mediated through the 5-HT system.^18^ Interestingly, in *Tph2*^−/−^ mice, which showed highest fear levels and flight responses to the FS, BL activity was not able to exceed home cage BL activity, while *Tph2*^+/−^ mice showed the strongest increase in BL c-Fos activity due to FS. No genotype effects were detected on the LA, providing evidence that the initial integration of FS with an auditory stimulus^42^ is 5-HT independent. As revealed by eIPSCs and paired-pulse ratio data, GABAergic transmission efficiency in the BL of *Tph2*^−/−^ and *Tph2*^+/−^ mice is reduced probably involving altered presynaptic release mechanism. This is in line with the view that 5-HT facilitates activity-dependent release of GABA via 5-HT2A receptors in distinct subtypes of GABAergic interneurons^43, 44^ in the BL. Thus, reduced efficacy of GABAergic transmission in *Tph2*^*+/−*^ mice seems to increase excitability of PNs in the BL, thereby functionally compensating a reduced 5-HT synthesis in *Tph2^+/−^* mice.

Especially the anterior BL has been shown to be part of a panic inhibition system^45^ that attributes emotional salience to both rewarding and aversive stimuli. Our results point towards the same neurons being activated, which are involved in the regulation of behavioral responses to a fear-related stimulus in an aversive OFT.^46, 47^ However, *Tph2*^−/−^ mice seem to lack the ability to increase anterior BL activity in order to inhibit flight responses in aversive and inescapable situations. Early life dysregulation of 5-HT signaling has been shown to affect both the number and regional distribution of interneuron subtypes in cortex^48, 49, 50^ and the BLC,^22^ it may well be that the excitability of the BL in *Tph2*^−/−^ mice is already saturated under control conditions. This might indicate an altered neuronal network in the BL of *Tph2*^−/−^ mice, which is unable to activate the panic-inhibition system as observed in *Tph2*^*+/−*^ mice, despite reduced efficacy of GABAergic transmission. This may explain the increased activity accompanied by increased panic-like undirected escape responses. Still, 5-HT deficiency may also affect other brain areas involved in the regulation of escape responses like the dorsolateral periaqueductal gray.^46, 51^ Thus, a dysfunctional regulation through lack of 5-HT within the periaqueductal gray following FS may explain increased freezing and escape responses in *Tph2*^−/−^ mice as well.

With respect to the fact that similar pathways seem to be involved in mediating the aversive inescapable nature of a context in the FS paradigm as well as in aversive and inescapable situations without reinforcement, such as the open field.^46^ *Tph2*^−/−^ mice seem to display more active coping, namely increased locomotor activity accompanied by escape-oriented panic-like behaviors. These data are in line with previous findings that blockade of 5-HT release from dopamine-sensitive 5-HT neurons in the DRD/DRV may be sufficient to produce a hyperlocomotion phenotype in the OFT.^52^ The anxiety-like state in open aversive inescapable contexts is reminiscent of distinct syndromal dimensions of PD; panic attacks occur unexpectedly, lacking an initiating cue, and rapidly precipitate avoidance of contexts.

Compared to fear, as observed in the freezing response to an inescapable FS or other aversive contexts, trait anxiety is a consequence of less definite, less expected or more distant threats.^7^ Different from fear, trait anxiety is not affected and seems even decreased by 5-HT deficiency, as observed in the LDT and NSFT.^53^ Similar to other models of 5-HT deficiency, social recognition were not affected by 5-HT deficiency as well.^54^ Of note, *Tph2*^−/−^ mice ignored a potentially dangerous context when a positive stimulus, such as food, was presented in the NSFT, suggesting a general failure of behavioral inhibition,^29, 52^ which may involve increased energy metabolism^55^ and food intake.^21, 55, 56^

In conclusion, exaggerated fear accompanied by a panic-like state and escape behavior resulting from lifelong absence of 5-HT synthesis involves dysfunction of the amygdalo-dorsal raphe circuitry controlling fear-related behavioral responses. This is due to alterations in GABAergic transmission prohibiting increased activity of the BL in aversive inescapable contexts. Increased escape responses reflect characteristic syndromal dimensions of panic disorder and agoraphobia. Thus, constitutive lack of 5-HT synthesis influences the risk for anxiety- and stressor-related disorders including panic disorder and comorbid agoraphobia through the absence of GABAergic-dependent compensatory mechanisms in the BLC.
