# Supplementary material for: Genetically driven brain serotonin deficiency facilitates panic-like escape behavior in mice
Source: Transl Psychiatry. 2017 Oct 3;7(10):e1246–. doi: 10.1038/tp.2017.209 (PMC5682603; doi:10.1038/tp.2017.209)
Supplement: Supplementary Methods [file tp2017209x1.docx]

**Behavioral analyses**

***Home cage activity***

To assess locomotor activity in a familiar environment, mice were observed in their home cages with lids and nesting material being removed. Cages were placed under a camera in a moderately illuminated room (100 lx) and animals were tracked for 30 min using VideoMot2 (TSE Systems, Bad Homburg, Germany). Variables measured included distance traveled, time spent moving and velocity.

***Light/dark box***

The light/dark box (LDB) consisted of a transparent Perspex ‘light’ compartment (40x40x27cm) and a black opaque Perspex ‘dark’ compartment (40x20x27cm). The dark chamber contained a small opening at floor level (5x5cm) and was covered by a removable lid. The illumination in the dark compartment was between 0 and 10 lx, whereas illumination in the light compartment was approximately 100 lx. Mice were placed in the dark section of the box and behavior was automatically recorded with VideoMot2 (TSE Systems, Bad Homburg, Germany) for 10 min. The latency to enter the lit compartment as well as the distance traveled and the time spent in the lit compartment were measured to determine anxiety-like behavior.

***Open-field test***

The open-field (OF) consisted of a black quadratic box (50x50x40 cm) semi-permeable to infrared light (Post et al., 2011). Illumination at floor level was between 50 and 100 lx (from the walls to the center of the arena). The arena was divided into a 30x30 cm center zone and the surrounding periphery. Mice were individually placed in one corner of the arena and automatically recorded for 30 min using VideoMot2 (TSE Systems, Bad Homburg, Germany). The distance traveled, time spent moving and velocity were used to evaluate locomotor activity; the number of center crossings and the time spent in the center of the arena were measured to determine anxiety-like behavior.

***Sociability and preference for social novelty***

The test apparatus was a black quadratic open-field (50x50x40 cm) semi-permeable to infrared light. Mice were habituated to the chamber during the 30 min OF test, which was performed three days before the two-trial social interaction test. In the first trial (sociability test), mice were given a choice between exploring a small empty wire cage or an identical enclosure containing an unfamiliar mouse. The small cages were located in the upper left and upper right corner of the OF and the position of the cage containing the stranger mouse vs. the empty cage was randomly alternated between animals to prevent possible effects of side preference. In the second trial (preference for social novelty test), mice were given a choice between exploring the first, now familiar mouse or a novel, unfamiliar mouse. The behavior in both 10 min trials was recorded using VideoMot2 (TSE Systems, Bad Homburg, Germany). The distance traveled, number of visits and time spent in each interaction zone (empty vs. mouse and familiar vs. novel, respectively) were measured to evaluate social interaction and social memory.

***Marble burying***

The marble burying test is a common test to assess anxiety- and compulsive-like behaviors. Mice were individually placed into Makrolon type III cages (42.5x26.6x15.5 cm) filled with clean bedding to a depth of 3 cm and 15 identical glass marbles that were evenly spaced across the bedding surface. After 30 min, mice were returned to the home cage and all marbles covered with bedding to two-thirds or more were scored as buried.

***Novelty-suppressed feeding***

The novelty-suppressed feeding (NSF) test is based on a conflict between food-seeking induced by hunger and the aversion to explore a novel, brightly illuminated environment (Dulawa and Hen, 2005). Food-deprived mice were placed in a corner of a white quadratic arena (50x50x40 cm; ~100 lx) containing a single food pellet (~2.5 g) on a petri dish located in the center of the box. After a test duration of 5 min, mice were returned to their home cage and allowed to eat a pre-weighed food pellet for 5 min. The latency to feed and the total feeding time in the novel environment were recorded as measures of anxiety-like behavior. The amount of food consumed in the home cage and the body weight loss during the food deprivation period (~14 h) were calculated to determine the animal’s appetitive drive.

**Immunhistochemical analysis**

Two hours after fear conditioning training brains were fixed by direct transcardial perfusion with ice-cold fixative I (4% paraformaldehyde (PFA); 2% sodium acetate (NaAc); pH 6.5) for 10 min followed by a second ice-cold fixative (fixative solution II: 4% PFA; 1.3% sodium carbonate (Na_2_CO_3_), 1.35% sodium bicarbonate (NaHCO_3_); 0.02% glutaraldehyde, pH 11) for 10 minutes. Post-fixation was conducted for 48 h in fixative solution II without glutaraldehyde and cryopreserved in 20% sucrose, then frozen in isopentane cooled with dry ice. Serially cut 30 µm cryostat sections spaced 180 µm apart were air-dried for 30 minutes at room temperature (RT) before heat-induced epitope retrieval in 10 mM citrate buffer (pH 6.0) containing 0.05% Tween-20 (pH 6). Nonspecific binding sites were saturated with a blocking solution (BS) containing 5% normal goat serum (NGS), 2% fetal bovine serum (FBS) and 0.25% Triton X-100 in Tris-buffered saline (TBS), incubated at 80 °C for 10 min. After cooling for 20 min and 3 x 5 min washes with TBS the sections were incubated in BS at RT for one hour. Primary antibodies were mouse anti-pyramidal (pyr) marker (1:200, Cat. No. 345, Swant, Marly, Switzerland), mouse anti-parvalbumin (PV) (1:200, Cat. No. 235, Swant, Marly, Switzerland) and rabbit anti-c-Fos (1:400, Cat. No. SC-52; Santa Cruz Biotechnology, Dallas, Texas, USA) diluted in BS and incubated over two nights at 4 °C in a humidified box. After washing away primary antibodies (3 x 5 min) with TBS, sections were incubated in 1:400 diluted secondary antibodies, goat anti-rabbit 555 (Cat. No. A21429, Invitrogen, Carlsbad, California, USA) and goat anti-mouse 488 (Cat.No. A11029, Invitrogen) diluted in BS for 1.5 hours at RT. After another (3 x 5 min) washing, the slides were treated with 300 nM DAPI (4', 6-diamidino-2-phenylindole) for 5 minutes and washed again. Finally the slides were washed in ddH_2_O to remove solutes, embedded with Fluoro-Gel (Cat. No.17985-10, EMS, Hatfield, PA, USA) and sealed with nail polish for long-term storage.

For the amygdala BLA and LA 3-4 consecutive sections from -1.22 mm bregma to -1.82 mm bregma, spaced 180μm apart, were delineated with contours according to the mouse brain atlas (Franklin and Paxinos, 1997).

For the dorsal hippocampus, the dentate gyrus (DG), Cornu Ammonis area 1 (CA1) as well as Cornu Ammonis area 3 (CA3), 4-5 consecutive sections from -1.06 mm bregma to -2.06 mm bregma, spaced 180μm apart, were delineated with contours according to the mouse brain atlas (Franklin and Paxinos, 1997).

Cells were counted as c-Fos-ir only when the nucleus showed complete fluorescent signal by a blinded researcher. The sum of counted cells of all sections per mouse were divided by the total contour area to calculate the cell densities per region of interest. Outliers were identified using the Grubbs’ test (Grubbs, 1969) and removed. For analysis of immunohistochemical staining, total cell densities were evaluated using GraphPad Prism version 6.00 (GraphPad Software, San Diego, California, USA).

**Electrophysiology in amygdala**

Five to six month old *Tph2*^-/-^, *Tph2*^+/-^ or *Tph2*^+/+^ mice were anaesthetized with Isoflurane (1-Chloro-2,2,2-trifluoroethyl-difluoromethylether; 2.5% in O_2_; Abbot GmbH & Co. KG, Germany) and decapitated. Coronal slices (300 µm thickness) containing the amygdala were prepared on a vibratome (Leica VT1200S), incubated at 32°C for 20 min and stored thereafter at room temperature (RT). Single slices were placed at RT in a submersion chamber and were perfused with artificial-cerebrospinal fluid (ACSF) containing [in mM]: NaCl 120, KCl 2.5, NaH_2_PO_4_ 1.25, MgSO_4_ 2, CaCl_2_ 2, NaHCO_3_ 22 and glucose 20. The pH was set to 7.35 by gassing with carbogen (95% O_2_, 5%CO_2_).

Whole-cell patch clamp recordings were performed using an EPC-10 patch-clamp amplifier (HEKA, Germany) at a sampling rate of 10 kHz. The recordings were done on principal-neurons in the LA, which were morphologically and electrophysiologically identified as previously described (Lange et al., 2014).

*Miniature (m), spontaneouse (s) and evoked (e) inhibitory postsynaptic currents (IPSCs):* Patch-pipettes (2.2-2,5 MΩ; borosilicate glass; Clark Electromedical Instruments, UK) were filled as follows [in mM]: NaCl 10, KCl 110, EDTA 11, HEPES 10, MgCl_2_ 1, CaCl_2_ 0,5, Phosphocreatin 15, MgATP 3, and NaGTP 0.5. The pH was set to 7.25. The isolated IPSCs were recorded in voltage-clamp mode at a membran potential of -70 mV in the presence of AP-5 (D-(-)-2-Amino-5-phosphonopentanoic acid; 50 µM; Abcam) and DNQX (6,7-Dinitroquinoxaline-2,3-dione disodium salt; 10 µM; Abcam) to block NMDA- and AMPA-receptors; mIPSCs were recorded in the presence of tetrodotoxin (TTX 1 µM).

The resting membrane potential was obtained immediately after accessing the whole-cell configuration. The passive and active membrane properties were recorded in the current-clamp mode at a membrane potential of -70 mV. Hyper- and depolarizing currents were injected (1000 ms duration; -60 pA first step; Δ+10 pA) to analyse passive and active membran properties. The input resistance was calculated from the steady-state voltage deflection in response to a hyperpolarizing current injection of -40 pA.

Amplitudes of eIPSCs were measured and averaged from 5 consecutive PSCs with an interval of 15 s. Obtained values were averaged from different neurons and are presented as the mean amplitude (in pA). The paired-pulse ratio of eIPSCs was calculated by dividing the amplitude of the second response by the amplitude of the ﬁrst responses at a paired-pulse interval of 50 ms. The mIPSCs and sIPSCs were recorded over a time period of 300 s (60 traces with 5 s duration) to determine amplitudes and frequencies of GABAergic postsynaptic currents.

**Slc6a4 *in situ* hybridization histochemistry**

Brains were sectioned throughout the midbrain and pons using a Leica 1900 cryostat (North Central Instruments, Plymouth, MN, USA; 5 alternate sets of 12 µm sections). Brain tissue sections were mounted onto 75 mm x 25 mm x 1 mm, positively-charged VWR Vistavision™ Histobond® microscope slides (16004-406, VWR International, Radnor, PA, USA) and stored at ‒80 °C. For semi-quantitative analysis of *Slc6a4* mRNA expression, gray values of digital autoradiography images were analyzed with ImageJ (NIH, Bethesda, MD, USA).

For *in situ* hybridization histochemical analysis of *Slc6a4* mRNA expression in the dorsal and median raphe nuclei, one oligonucleotide probe complementary and specific to *Slc6a4* mRNA (5’- ACTGCAGAGTACCCATTGGATATTTGGCTAGGCTCTGCCCTGTCCGCTGT-3’) was transcribed (Integrated DNA Technologies). Following transcription, probe was radiolabeled with [35-S]-deoxyadenosine-5’-(alpha-thio)-triphosphate (dATP, Cat. No. NEG034H001MC, PerkinElmer) using the enzyme terminal transferase (Promega) for 1 hr at 37 °C in a water bath to create a DNA oligonucleotide probe complementary to bases 207-256 of murine *Slc6a4* mRNA (Hansen and Mikkelson, 1998).

The oligonucleotide probes were then cleaned (QIAquick Nucleotide Removal® kit, Cat. No. 28304, Qiagen, Valencia, CA, USA), and *in situ* hybridization histochemistry was performed as previously described (Gardner et al., 2009). Briefly, slides were equilibrated to room temperature and then immersed in 4% paraformaldehyde in 0.05 M phosphate buffered saline (PBS) for 10 min. Following two washes in 0.05 M PBS, slides were placed into freshly prepared 0.25% acetic anhydride in 0.9% NaCl containing 0.1 M triethanolamine (TEA) for 10 min. Sections were then dehydrated through a graded series of alcohol washes, delipidated in chloroform, rehydrated through a second series of alcohol washes, and then allowed to air dry.

Oligonucleotide probe hybridization solution (50% formamide, 20 × standard saline citrate (SSC), 25 mg/ml yeast tRNA, 10 mg/ml sheared salmon sperm DNA, 50X Denhardt’s solution, 50% dextran sulphate, 10 mM dithiothreitol (DTT) and 1 × 10^6^ cpm total radiolabeled probe) was placed on each slide (90 μl), covered with Parafilm M® coverslips, and incubated overnight in a humidified 37 °C chamber. The next day coverslips were removed in 1X SSC and each slide was washed with agitation 4 × 15 sec in 1X SSC. Slides were then put through 4 × 15 min washes in 1X SSC at 55 °C in a shaking water bath, 1X SSC at room temperature for 2 × 30 min and then briefly (1–2 sec) in distilled water at room temperature. Slides were then air-dried, and apposed onto a BioMax MR autoradiography film (Cat. No. 871 5187, Carestream Health, Rochester, NY, USA) along with ^14^C standards (Cat. No. ARC0146C, American Radiolabeled Chemicals, Inc., St. Louis, MO, USA) for a period 36 days (*Slc6a4*).

**Semi-quantitative analysis of Slc6a4 mRNA expression**

For semi-quantitative analysis of *Slc6a4* mRNA expression, digital autoradiography images were analyzed with ImageJ (NIH, Bethesda, MD, USA), while the researcher was blinded to the treatment groups to measure gray value x area (as a measure of mRNA expression) using matrices in the shape of each subdivision of the brainstem DR or MnR. Area (mm^2^) was defined as the area (within each matrix) that fell above a gray value threshold that was kept consistent throughout analysis. Based on Gardner et al 2009, a total of 12 rostrocaudal sections, designated levels +6 to −5, containing 7 major subdivisions of the DR were analyzed (dorsal raphe nucleus, dorsal part, DRD, −4.244 to −4.644 mm bregma; dorsal raphe nucleus, ventral part, DRV, −4.244 to −4.724 mm bregma; dorsal raphe nucleus, ventrolateral part/ventrolateral periaqueductal gray region, DRVL/VLPAG, −4.544 to −4.724 mm bregma; dorsal raphe nucleus, caudal part, DRC, −4.724 to −4.904 mm bregma; dorsal raphe nucleus, interfascicular part, DRI, −4.784 to −4.904 mm bregma) (Paxinos & Franklin, 2003). An average value was computed for the DRVL/VLPAG using values from both the left and right hemisphere. The MnR was examined from −4.544 mm −4.644 mm bregma. Background measurements were taken in the lateral periaqueductal gray. The average background gray value for each section was subtracted from the mean gray value for the above threshold pixels for each subregion; the resulting number was then multiplied by the above-threshold area of the subregion (in mm^2^) to generate a semi-quantitative measurement of gene expression within that region. An atlas of *Slc6a4* (Fig. S3) mRNA expression was used to aid in keeping the analysis consistent.

For statistical comparisons, the software package SPSS (version 22.0, SPSS Inc., Chicago, IL, USA) was used. Average levels of gene expression for each DR subdivision at each rostrocaudal level of the DR in each treatment group were generated. Outliers were identified using the Grubbs’ test (Grubbs, 1969) and removed. Following outlier removal, an overall linear mixed model (LMM) with repeated measures (subregion and rostrocaudal level, with rostrocaudal level nested within subregion) was used to detect overall fixed effects of genotype, raphe subregion, rostrocaudal level and interactions among these factors (Tables S1 and S2). Following this, additional LMMs were conducted on subsets of the data containing only data from individual DR subdivisions to determine the locations of effects identified in the overall model. An autoregressive heterogeneous covariance structure was specified for all LMMs. Individual pairwise comparisons between genotypes were made using Fisher’s protected least significant difference tests, the significance was set at *p* < 0.05.
