# Supplementary material for: Genetically driven brain serotonin deficiency facilitates panic-like escape behavior in mice
Source: Transl Psychiatry. 2017 Oct 3;7(10):e1246–. doi: 10.1038/tp.2017.209 (PMC5682603; doi:10.1038/tp.2017.209)
Supplement: Supplementary Figures [file tp2017209x2.pdf]

Fig. S1

a) Light-dark box

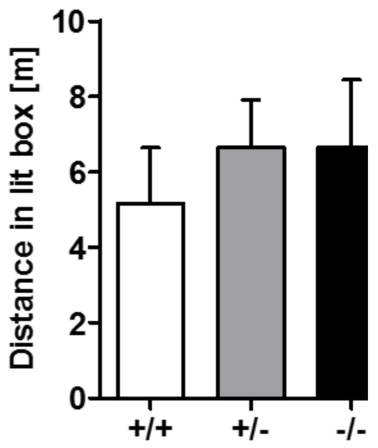


b) Open-field

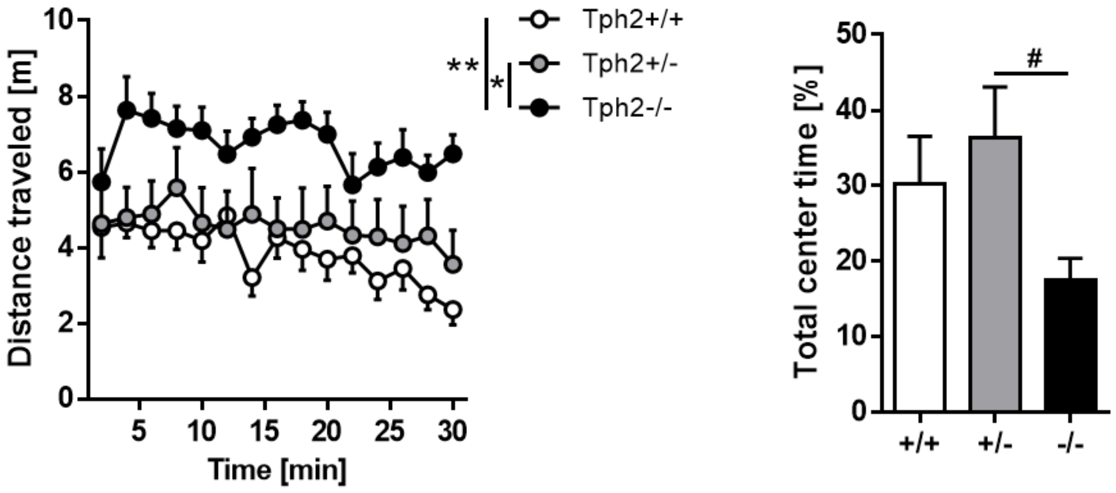

Fig. S2

a) Marble burying

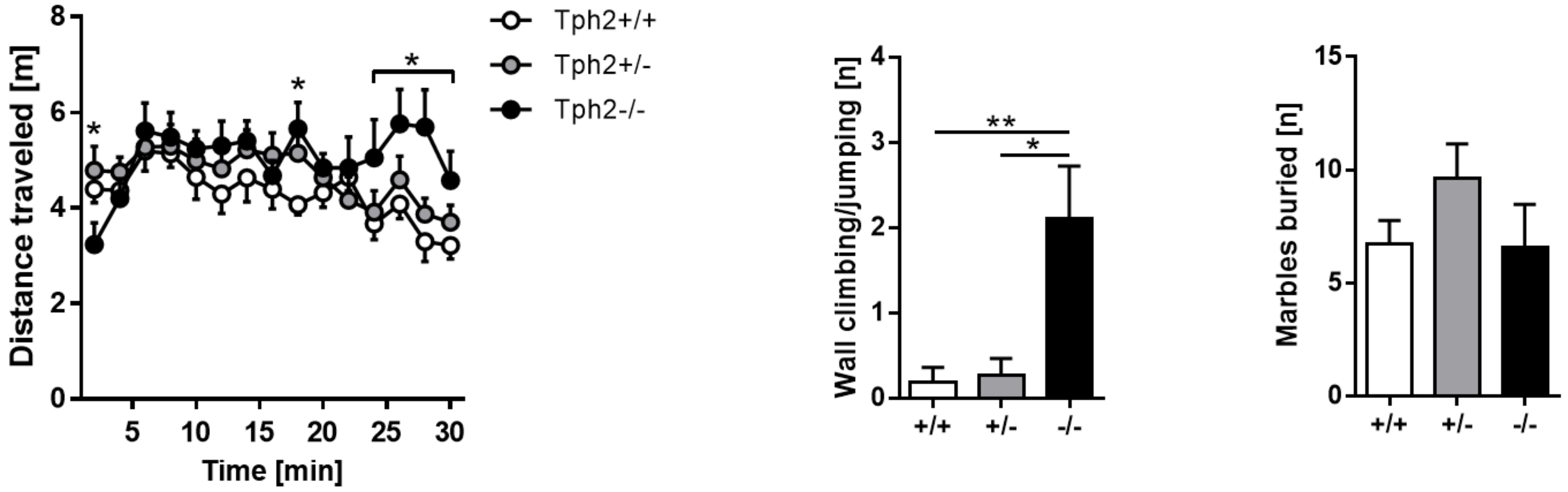


b) Social interaction

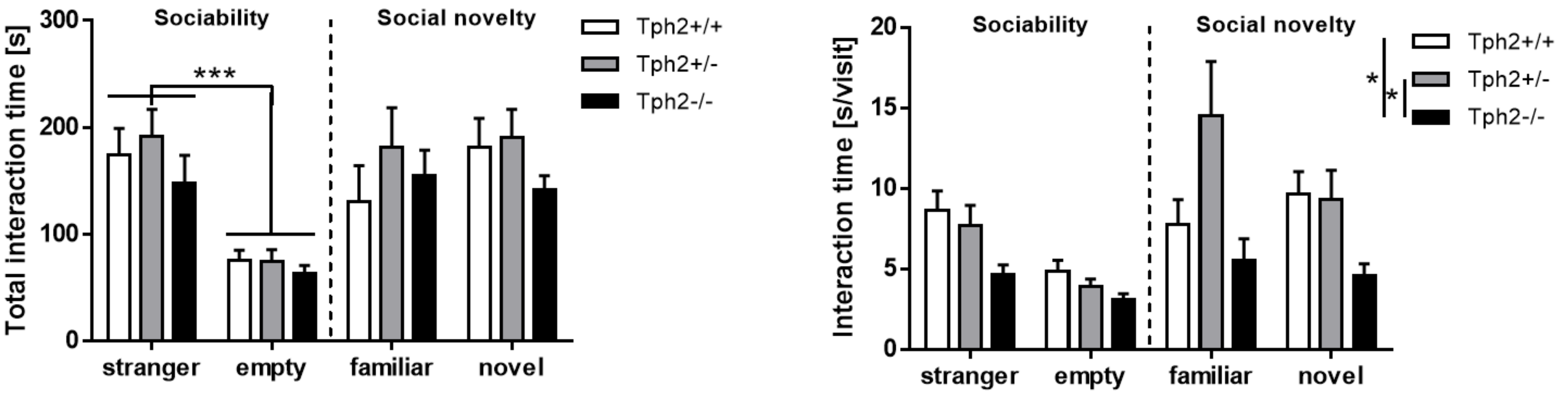

Fig. S3

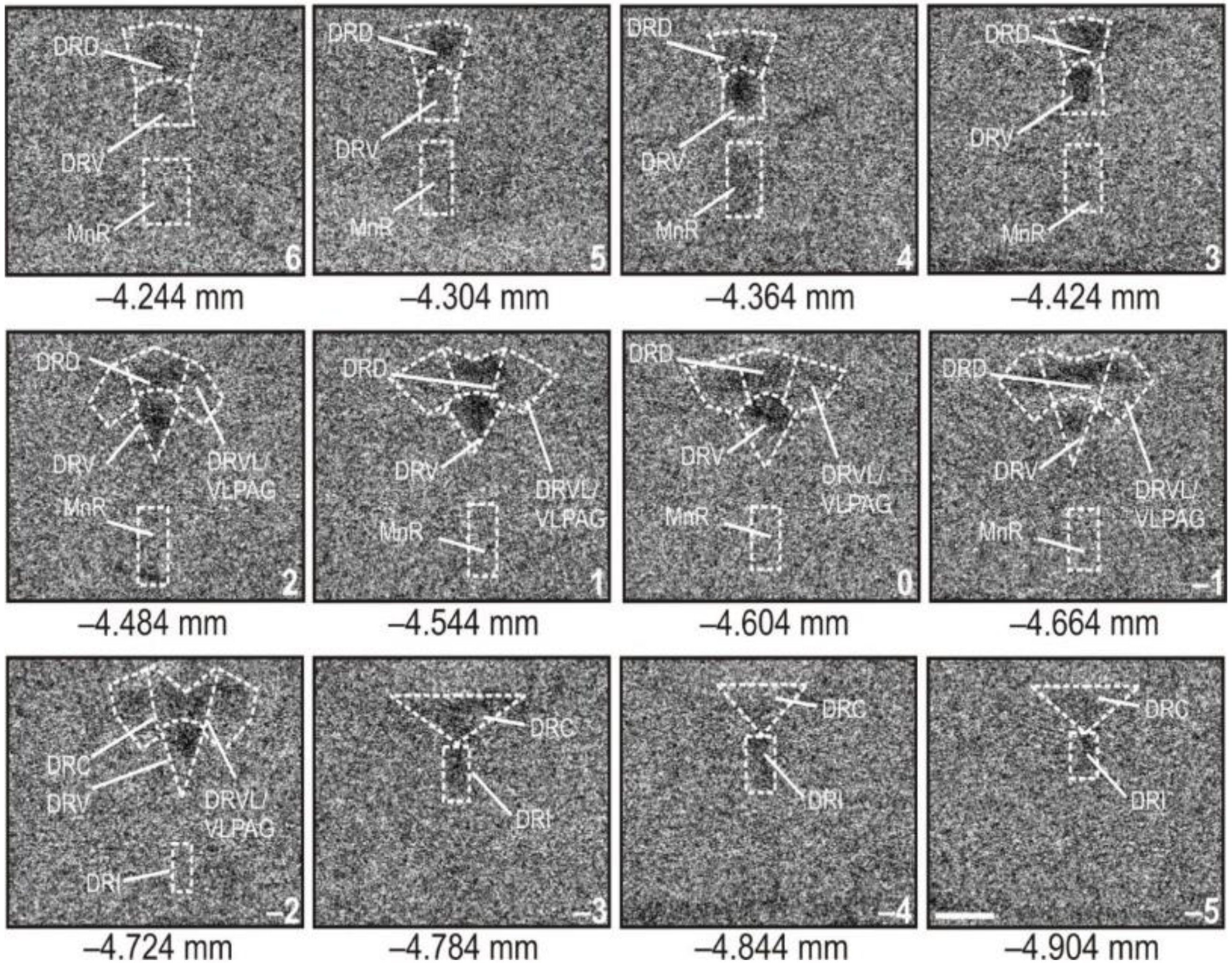

DRD
DRV
MnR
6
–4.244 mm
DRD
DRV
MnR
5
–4.304 mm
DRD
DRV
MnR
4
–4.364 mm
DRD
DRV
MnR
3
–4.424 mm
DRD
DRV
DRVL/
VLPAG
MnR
2
–4.484 mm
DRD
DRV
DRVL/
VLPAG
MnR
1
–4.544 mm
DRD
DRV
DRVL/
VLPAG
MnR
0
–4.604 mm
DRD
DRV
DRVL/
VLPAG
MnR
–1
–4.664 mm
DRC
DRV
DRVL/
VLPAG
DRI
–2
–4.724 mm
DRC
DRI
–3
–4.784 mm
DRC
DRI
–4
–4.844 mm
DRC
DRI
–5
–4.904 mm

Fig. S4

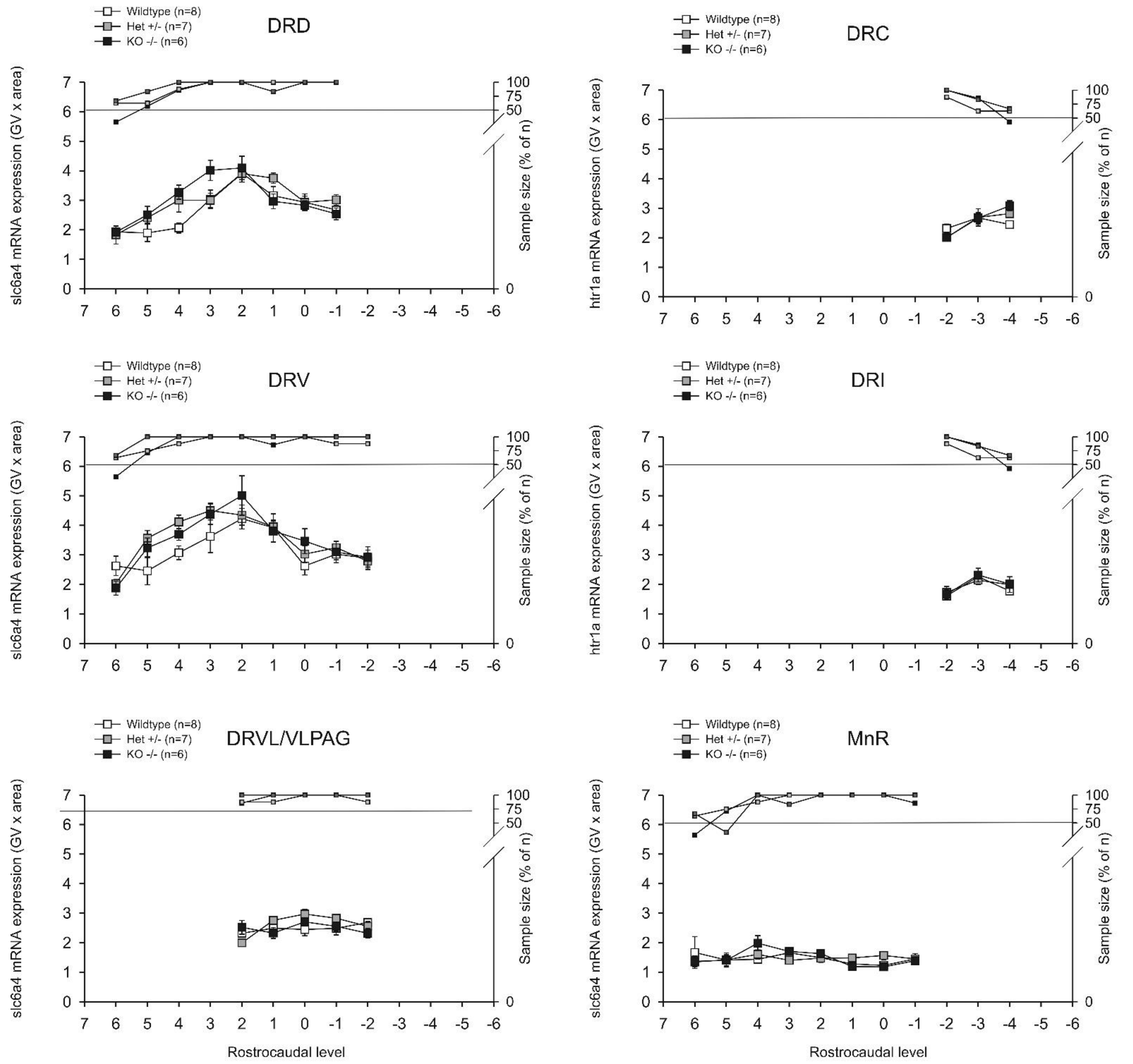
Wildtype (n=8)
Het +/- (n=7)
KO -/- (n=6)
DRD
DRC
DRV
DRI
DRVL/VLPAG
MnR
slc6a4 mRNA expression (GV x area)
htr1a mRNA expression (GV x area)
Sample size (% of n)
Rostrocaudal level

**Table S1**: Fixed effects of the Linear Mixed Models used to analyze *slc6a4* mRNA expression

| Model | Source | Test statistic | *p*-value |
|---|---|---|---|
| **Overall Analysis** | | | |
| **Entire dataset** | | | |
| | Genotype | $F_{(2, 152.1)} = 8.9$ | 0.001 |
| | Raphe subregion | $F_{(6, 119.8)} = 533.6$ | 0.001 |
| | Rostrocaudal level | $F_{(10, 102.4)} = 51.8$ | 0.001 |
| | Genotype*Rostrocaudal level(Raphe subregion) | $F_{(122, 56.2)} = 7.1$ | 0.001 |
| **Subregional Analyses** | | | |
| **Mean *slc6a4* mRNA expression in entire DR** | | | |
| | Genotype | $F_{(2, 51.7)} = 4.3$ | 0.019 |
| | Rostrocaudal level | $F_{(10, 30.8)} = 58.3$ | 0.001 |
| | Genotype*Rostrocaudal Level | $F_{(20, 30.9)} = 1.536$ | 0.138 |
| **DRD** | | | |
| | Genotype | $F_{(2, 40.9)} = 2.4$ | 0.060 |
| | Rostrocaudal level | $F_{(7, 28.0)} = 15.7$ | 0.001 |
| | Genotype*Rostrocaudal Level | $F_{(14, 31.3)} = 1.5$ | 0.156 |
| **DRV** | | | |
| | Genotype | $F_{(2, 38.2)} = 3.0$ | 0.107 |
| | Rostrocaudal level | $F_{(8, 32.5)} = 15.3$ | 0.001 |
| | Genotype*Rostrocaudal Level | $F_{(16, 32.6)} = 1.5$ | 0.158 |
| **DRVL/VLPAG** | | | |
| | Genotype | $F_{(2, 28.9)} = 1.1$ | 0.363 |
| | Rostrocaudal level | $F_{(4, 30.4)} = 3.3$ | 0.024 |
| | Genotype*Rostrocaudal Level | $F_{(8, 30.5)} = 2.7$ | 0.024 |
| **DRC** | | | |
| | Genotype | $F_{(2, 16.3)} = .3$ | 0.749 |
| | Rostrocaudal level | $F_{(2, 24.4)} = 22.3$ | 0.001 |
| | Genotype*Rostrocaudal Level | $F_{(4, 26.4)} = 3.3$ | 0.025 |
| **DRI** | | | |
| | Genotype | $F_{(2, 16.7)} = .3$ | 0.761 |
| | Rostrocaudal level | $F_{(2, 16.1)} = 9.1$ | 0.002 |
| | Genotype*Rostrocaudal Level | $F_{(4, 16.3)} = .3$ | 0.893 |
| **MnR** | | | |
| | Genotype | $F_{(2, 35.3)} = .1$ | 0.94 |
| | Rostrocaudal level | $F_{(7, 28.9)} = 3.2$ | 0.013 |
| | Genotype*Rostrocaudal Level | $F_{(14, 25.8)} = 1.7$ | 0.112 |
