# Supplementary material for: Genetically driven brain serotonin deficiency facilitates panic-like escape behavior in mice
Source: Transl Psychiatry. 2017 Oct 3;7(10):e1246–. doi: 10.1038/tp.2017.209 (PMC5682603; doi:10.1038/tp.2017.209)
Supplement: Supplementary Figure legends [file tp2017209x3.docx]

**Supplementary Figures**

Suppl Fig 1. Anxiety-like behaviors of *Tph2* deficient mice. (a) Distance traveled in lit compartment in the light-dark transition test. (b, left panel) Time course of distance traveled. (b, right panel) Total center time in the open-field test. Data are shown as means ± SEM. ^#^ *p*<0.1, **p*<0.05 and ***p*<0.01 compared to respective controls.

Suppl Fig 2. Marble burying and social interaction of *Tph2* deficient mice.

(a, left panel) Time course of distances traveled of *Tph2^-/-^* , *Tph2^+/-^* and *Tph2^+/+^* mice in a marble burying task as well as (a, middle panel) number of wall jumps and (a, right panel) number of marbles buried. (b, left panel) Total interaction time in a two-trial social interaction test as well as sociability (interaction time with an unfamiliar mouse vs. an empty cage) and (b, right panel) preference for social novelty (interaction time with a novel conspecific vs. a familiar mouse) (b) Data are shown as means ± SEM. ^#^ *p*<0.1, **p*<0.05, ***p*<0.01 and ****p*<0.001 compared to respective controls.

Suppl Fig 3. Atlas used to analyze *slc6a4* (solute carrier family 6, member 4) mRNA expression in subregions of the murine dorsal raphe nucleus and median raphe nucleus. Borders of each subregion are denoted by white dashed lines. Rostrocaudal location of each section is shown below each photomicrograph in millimeters from bregma. The white numbers in the bottom right of each panel indicate experimenter designated rostrocaudal level used in analysis. Abbreviations: dorsal raphe nucleus, caudal part (DRC);dorsal raphe nucleus, dorsal part (DRD); dorsal raphe nucleus interfascicular part (DRI); dorsal raphe nucleus, ventral part (DRV); dorsal raphe nucleus, ventrolateral part/ventrolateral periaqueductal gray region (DRVL/VLPAG); median raphe nucleus (MnR). Scale bar, 250 μm.

Suppl Fig 4. *Slc6a4* (solute carrier family 6, member 4) mRNA expression throughout the rostrocaudal extent of subregions of the murine dorsal raphe nucleus and median raphe nucleus. The left y axis indicates slc6a4 mRNA expression expressed as gray value (GV) x area. The right y axis indicates sample sizes, expressed as percent of the total, upon which each data point is determined. Values represent means ± SEM. Abbreviations: dorsal raphe nucleus, caudal part (DRC);dorsal raphe nucleus, dorsal part (DRD); dorsal raphe nucleus interfascicular part (DRI); dorsal raphe nucleus, ventral part (DRV); dorsal raphe nucleus, ventrolateral part/ventrolateral periaqueductal gray region (DRVL/VLPAG); median raphe nucleus (MnR).
